# A pilot study for risk stratification of ventricular tachyarrhythmia in hypertrophic cardiomyopathy with routine echocardiography parameters

**DOI:** 10.1038/s41598-024-54153-2

**Published:** 2024-02-15

**Authors:** Anselm A. Derda, Malin Abelmann, Jan-Thorben Sieweke, Florian J. G. Waleczek, Natalie Weber, Nadine Zehrfeld, Christian Bär, David Duncker, Udo Bavendiek, Dominik Berliner, Johann Bauersachs, Kristina Sonnenschein, Thomas Thum

**Affiliations:** 1 Department of Cardiology and Angiology, Hannover Medical School, Hannover, Germany; 2 Institute of Molecular and Translational Therapeutic Strategies (IMTTS), Hannover Medical School, Carl-Neuberg-Str. 1, 30625 Hannover, Germany; 3 Fraunhofer Institute of Toxicology and Experimental Medicine, Hannover, Germany; 4 Department of Rheumatology and Immunology, Hannover Medical School, Hannover, Germany

**Keywords:** Cardiac hypertrophy, Ventricular tachycardia

## Abstract

Ventricular tachyarrhythmia (VTA) are frequent arrhythmias in patients with hypertrophic cardiomyopathy (HCM). Representing a major risk factor for sudden cardiac death, Holter ECG at first clinical presentation appears insufficient. This study aims to investigate the ability of routinely obtained parameters associated with myocardial remodeling in stratifying for VTA in HCM. In this monocentric analysis, patients with HCM underwent 12-channel electrocardiography and echocardiography, including tissue doppler imaging. The study’s primary endpoint was the documentation of non-sustained and sustained ventricular tachycardia—summarized as ventricular tachyarrhythmias (VTA) on Holter ECG or active devices. The occurrence of VTA was exploratory. Based on our collective, we developed a risk model regarding VTA. Of 140 HCM patients, 38 (27.1%) had an episode of VTA. Patients with VTA were likelier to have a history of atrial fibrillation (p < 0.001), a thicker interventricular septum (p < 0.001) and lower peak systolic mitral annular velocity (p < 0.001). The parameters were independently associated with endpoint in univariate and multivariate logistic regression. We created a logistic equation and calculated a cut-off value. The resulting ROC curve revealed a discriminative ability with AUC of 0.80 (sensitivity, 63%; specificity, 88%). Our risk model including these widely available parameters is able to distinguish low and high-risk of VTA in patients with HCM.

## Introduction

Hypertrophic cardiomyopathy (HCM) is the most frequent inherited cardiovascular disorder in adults with a reported prevalence of up to 1 in 200 in the general population^1^. It is a genetic disease with a complex phenotype caused by mutations in more than a dozen genes mostly encoding sarcomere proteins, which lead to hypertrophy, fibrosis, and myocyte disarray^2^. These histological changes are substrates for the increased risk to develop ventricular arrhythmia, such as sustained or non-sustained ventricular tachycardia—summarized as ventricular tachyarrhythmias (VTA)^2^. Sudden cardiac death (SCD) is a feared complication, especially in young adults^3^. To assess the risk of SCD a risk calculator is recommended by the European Society of Cardiology (ESC)^4^. Age, history of syncope, various echocardiographic parameters (maximum left ventricular wall thickness, left atrial size, and maximum gradient of the left ventricular outflow tract), and ventricular tachycardia play an essential role in risk prediction^4^. The 48-h Holter ECG is the current standard for detecting VTA at initial clinical admission^5^. The ESC guidelines recommend a 48-h Holter ECG scan at the patient's initial presentation. However, ECG monitoring beyond 48 h may be required since ventricular tachycardia are often asymptomatic^6^. In our experience, patients who do not use specialty outpatient clinics or have limited proximity to urban centers with sound medical facilities tend to have infrequent access to Holter ECG or undergo such diagnostic procedures at inadequate intervals. We aim to identify HCM patients at high-risk for VTA, which may benefit from intensified monitoring via Holter ECG (even beyond 48 h) or event recorder. Previous studies have described that individual parameters in ECG^7,8^ or echocardiography^9,10^ may be associated with bad outcome in HCM, but no combination of parameters has ever been investigated regarding the occurrence of VTA.

## Methods

### Study design and participants

The study recruitment occurred between July 2011 and December 2022 at the special outpatient clinic for HCM of the Department of Cardiology and Angiology at Hannover Medical School (MHH). During their regular outpatient appointment, 140 patients were included in the study. The diagnosis of HCM was based on the recent ESC guidelines for the diagnosis and management of hypertrophic cardiomyopathies, which included patients with familial or genetically diagnosed HCM with wall thickness ≥ 13 mm in one or more left ventricular (LV) myocardial segments or patients with wall thickness ≥ 15 mm in the absence of any other cause for hypertrophy^5^. The following exclusion criteria were defined in advance: age < 18, moderate to severe aortic stenosis, amyloidosis, Fabry disease, and patients unable to provide informed consent. 12 patients were excluded because of incomplete acquisition of study data.

Each patient gave written informed consent. The study was approved by the ethics committee at Hannover Medical School (Ethics vote no.5632) and was conducted according to the ethical principles of the Declaration of Helsinki.

Baseline characteristics were collected by clinical records. At the presentation, each patient underwent a clinical examination, a 12-lead ECG, and a comprehensive echocardiographic examination. Following this, Holter ECG monitoring or interrogation of an implantable device was used to assess the primary endpoint. The primary endpoint was ventricular tachyarrhythmia (VTA) consisting of both sustained (sVT) and non-sustained (nsVT) forms. The nsVT was defined as ≥ 3 consecutive ventricular beats at a rate of ≥ 120 beats per minute and a duration of < 30 s according to the ESC guidelines for the diagnosis and management of hypertrophic cardiomyopathy^5^. In contrast, sVT lasted for 30 s or more^5^. In addition, for each patient, the ESC risk score, estimating the 5-year risk of SCD, was calculated.^4^.

### Echocardiography

Echocardiographic images were acquired using Phillips ultrasound systems. Single-blinded investigators unaware of VTA status performed echocardiographic analyses.

Using 2D images, the thickness of the interventricular septum during diastole (IVSd), left ventricular end-diastolic diameter (LVEDD) and LV posterior wall during diastole (LVPWd) were measured. For quantification of the left atrial size, two different measurements were utilized: the left atrial diameter in maximum expansion in the parasternal long axis (LA PLAX) and the left atrial volume index (LAVI) measured from the apical four- and two-chamber views and then indexed to body surface area^11^. The echocardiography was carried out according to the recommendations of the American Society of Echocardiography^12^.

Different diastolic indices were evaluated using pulsed-wave Doppler: peak velocity of mitral E wave, peak velocity of the mitral A wave, ratio of mitral peak early to mitral peak late filling velocity (E/A) and deceleration time of the mitral E wave.

Using Tissue Doppler imaging, peak myocardial early (e′) and late (a′) diastolic velocity and peak systolic (s′) velocity were obtained in the apical four-chamber view at the medial and lateral mitral annulus at the highest possible frame rate. e′, a′ and s′ average were calculated by averaging medial and lateral values^13^. In 13 patients a′ average could not be measured because they were not in sinus rhythm during the echocardiogram. Transmitral early diastolic velocity ratio (E/e′ lateral, medial and average) was calculated for each patient.

### Statistical analysis

Categorical variables are presented as numbers and percentages, and continuous variables either as mean ± standard deviation (SD) for normally distributed variables or as median and interquartile ranges (IQR) for non-normally distributed variables. The distribution of continuous data of our entire HCM cohort was tested for normality using Kolmogorov–Smirnov and Shapiro–Wilk tests. Group differences of continuous data were analyzed using the two-sided Student's t-test for normally distributed data or the Mann–Whitney U-test for ordinal or non-normally distributed data. The distribution of categorical variables was analyzed using chi-square or Fisher's exact test. A comparison between patients with and without VTA was performed for the complete dataset.

### Risk stratification and score development

Binary logistic regression analysis was used to identify variables associated with the occurrence of VTA. Before multivariate analysis, multicollinearity was excluded. After checking for significant results in univariate analyses, multivariate logistic regression was performed. Backward elimination technique was used to find independently associated parameters with the development of VTA. Only variables with p < 0.05 were included in the final model. Goodness-of-fit was assessed by the Hosmer–Lemeshow test. Significant correlations between the predictors and other echocardiographic measurements were examined using Pearson correlation and t-test. Results from our regression analysis are presented as odds ratios (ORs) with 95% confidence intervals. Odds ratios and the beta coefficients used in the logistic equation can be converted into each other: OR = e^β^. To avoid overfitting, the number of potential determinants was limited to a maximum of three^14^.

In the end, three parameters (AF, IVSd, s′ average), independently associated with our endpoint, were included in the risk stratification model. The area under the curve (AUC) was calculated to evaluate the discriminatory ability of the risk stratification score.

All statistical analyses were performed with Statistical Package for the Social Science, version 28.0 (IBM SPSS, Armonk, NY, USA), and statistical significance was defined by a p-value < 0.05. Figures were created using GraphPad Prism 9.0.1 (GraphPad Inc., La Jolla, CA, USA) and Biorender.com.

### Statements and declarations

Each patient gave written informed consent. The study was approved by the ethics committee at Hannover Medical School (Ethics vote no.5632) and was conducted according to the ethical principles of the Declaration of Helsinki.

## Results

### Characteristics of the study cohort

**Figure 1:**
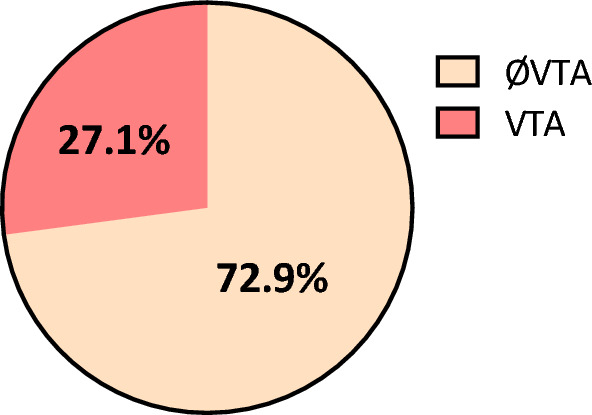
Ventricular tachyarrhythmia in our study cohort.

Baseline characteristics, including a comparison of demographic, echocardiographic and TDI data, are summarized in Table 1. 140 HCM patients with a median age of 57 (IQR: 45–66) years were included in the study. 59% of the study cohort were male. 38 patients (27.1%) reached the clinical endpoint (Fig. 1), of whom 36 patients (25.7%) developed nsVT, while only two patients (1.4%) developed sVT. Patients with VTA did not differ significantly from those without VTA regarding age, gender, BMI or LV outflow tract obstruction. Neither the diastolic nor the systolic blood pressure differed significantly between both groups. Furthermore, no substantial differences were detected regarding interventions such as myectomy or Transcoronary ablation of septal hypertrophy (TASH) (Table 1). Patients with VTA were more likely to suffer from palpitations, and to have an implanted cardioverter-defibrillator (ICD), although these values did not reach statistical significance. Likewise, no significant differences in family history of HCM or SCD, syncope and dyspnea, assessed with NYHA classification, could be observed. The groups did not significantly differ concerning cardiac medication at admission. For the mineralocorticoid receptor antagonist, there was a formally statistically significant difference (p = 0.043).

**Table 1:** Demographic and clinical characteristics of the study collective.

	Total (n = 140)	Patients with VTA (n = 38)	Patients without VTA (n = 102)	p-value
Demographics				
Age (years)	57 (45–66)	56 (45–64)	57 (45–66)	n.s.
Sex, male (%)	83 (59)	23 (61)	60 (59)	n.s.
BMI (kg/m^2^)	28 (25–31)	27 (24–31)	28 (25–32)	n.s.
HOCM (%)	81 (57)	17 (45)	64 (63)	n.s.
Systolic blood pressure (mmHg)	131 (120–140)	130 (120–140)	133 (121–140)	n.s.
Diastolic blood pressure (mmHg)	75 (66–80)	74 (68–85)	75 (65–80)	n.s.
ICD (%)	26 (19)	11 (29)	15 (15)	n.s.
Myectomy (%)	7 (5)	2 (5)	5 (5)	n.s.
TASH (%)	7 (5)	1 (3)	6 (6)	n.s.
HCM SCD risk score	2.6 (1.6–4.7)	5.1 (3.4–6.9)	2.1 (1.3–3.3)	< 0.001
Atrial fibrillation (%)	28 (20)	17 (45)	11 (11)	< 0.001
Family history of HCM	34 (24)	10 (26)	24 (24)	n.s.
Family history of SCD	15 (11)	5 (13)	10 (10)	n.s.
Medication at first admission				
Beta blocker (%)	93 (66)	29 (76)	64 (63)	n.s.
ACEi (%)	37 (26)	9 (24)	28 (27)	n.s.
ARB (%)	23 (16)	7 (18)	16 (16)	n.s.
MRA (%)	18 (13)	9 (24)	9 (9)	0.043
Diuretics (%)	44 (31)	13 (34)	31 (30)	n.s.
CCB DHP (%)	22 (16)	7 (18)	15 (15)	n.s.
CCB non-DHP (%)	18 (13)	5 (13)	13 (13)	n.s.
Electrocardiography				
PR (ms)^a^	160 (146–184)	162 (140–181)	158 (146–182)	n.s.
QRS (ms)	104 (95–116)	101 (95–112)	102 (92–115)	n.s.
QTc (ms)	444 (430–460)	449 (420–478)	444 (427–459)	n.s.
Symptoms				n.s.
NYHA	2 (1–2)	2 (1–2)	2 (1–2)	n.s.
Palpitations (%)	45 (32)	17 (45)	28 (28)	n.s.
Syncope (%)	18 (13)	5 (13)	13 (13)	n.s.
Echocardiography				
IVSd (mm)	19 (16–22)	22 (18–27)	18 (15–21)	< 0.001
LA PLAX (mm)	43 (36–50)	44 (36–52)	43 (36–50)	n.s.
LAVI (ml/m^2^)	41 (34–58)	43 (37–72)	39 (32–54)	0.015
LVEDD (mm)	43 (40–48)	43 (40–49)	42 (39–47)	n.s.
LVPWd (mm)	12 (10–14)	13 (10–13)	12 (10–14)	n.s.
LV Mass Index (g/m^2^)^b^	136 (103–157)	139 (111–155)	132 (100–156)	n.s.
E wave (cm/s)	77 (65–94)	80 (65–96)	74 (65–93)	n.s.
A wave^c^ (cm/s)	74 (54–90)	67 (47–89)	76 (56–92)	n.s.
Deceleration time (ms)	220 (161–270)	215 (133–282)	220 (170–270)	n.s.
E/A^c^	1.05 (0.77–1.35)	1.3 (0.9–1.4)	1.0 (0.7–1.4)	n.s.
E/e′ average	12.5 (9.8–16)	13 (10–17)	12 (9–16)	n.s.
s′ average (cm/s)	6.7 (5.8–7.7)	5.9 (4.9–6.9)	6.9 (6.1–8.0)	< 0.001
Medial s′ (cm/s)	6.5 (5.1–7.9)	5.7 (4.3–7.1)	6.8 (5.5–8.1)	< 0.001
Lateral s′ (cm/s)	6.8 (5.8–8.1)	6.4 (5.0–7.1)	7.1 (6.0–8.5)	0.003
e′ average (cm/s)	6.2 (4.8–7.8)	5.9 (4.7–7.9)	6.2 (4.8–7.7)	n.s.
Medial e′ (cm/s)	4.8 (3.9–6.3)	4.7 (3.5–6.8)	4.9 (3.9–6.0)	n.s.
Lateral e′ (cm/s)	7.2 (5.2–9.7)	6.7 (5.0–10.0)	7.3 (5.4–9.7)	n.s.
a′ average (cm/s)^c^	8.3 (6.9–9.9)	7.1 (5.8–9.1)	8.9 (7.4–10.3)	0.03
Medial a′ (cm/s)^c^	7.5 (6.3–9.2)	6.9 (5.6–8.1)	7.6 (6.5–9.3)	0.014
Lateral a′ (cm/s)^c^	8.8 (7.0–11.6)	7.1 (5.1–9.9)	9.1 (7.7–12.0)	0.006

BMI, body mass index; HOCM, hypertrophic obstructive cardiomyopathy; ICD, implantable cardioverter-defibrillator; TASH, transcoronary ablation of septal hypertrophy; ACEi, angiotensin converting enzyme inhibitor; ARB, angiotensin receptor blocker; MRA, mineralocorticoid receptor antagonist; CCB DHP, dihydropyridine calcium channel blocker; CCB Non-DHP, non-dihydropyridine calcium channel blocker; IVSd, interventricular septum in diastole; LA PLAX, left atrium in parasternal long axis; LAVI, left atrial volume index; LVEDD, left ventricular end diastolic diameter; LVPWd, left ventricular posterior wall at end diastole, LV Mass Index, left ventricular mass index.

^a^Data missing due to atrial fibrillation while electrocardiography or missing electrocardiography (n = 120).

^b^Data missing due to incomplete echocardiography (n = 104).

^c^Data missing due to atrial fibrillation while echocardiography (n = 127).

### ECG characteristics

HCM patients with VTA were more likely to have diagnosed atrial fibrillation (45% vs. 11% p < 0.001). The two groups did not significantly differ regarding ECG intervals and durations (PR, QRS or QTc).

### Echocardiographic characteristics

**Figure 2:**
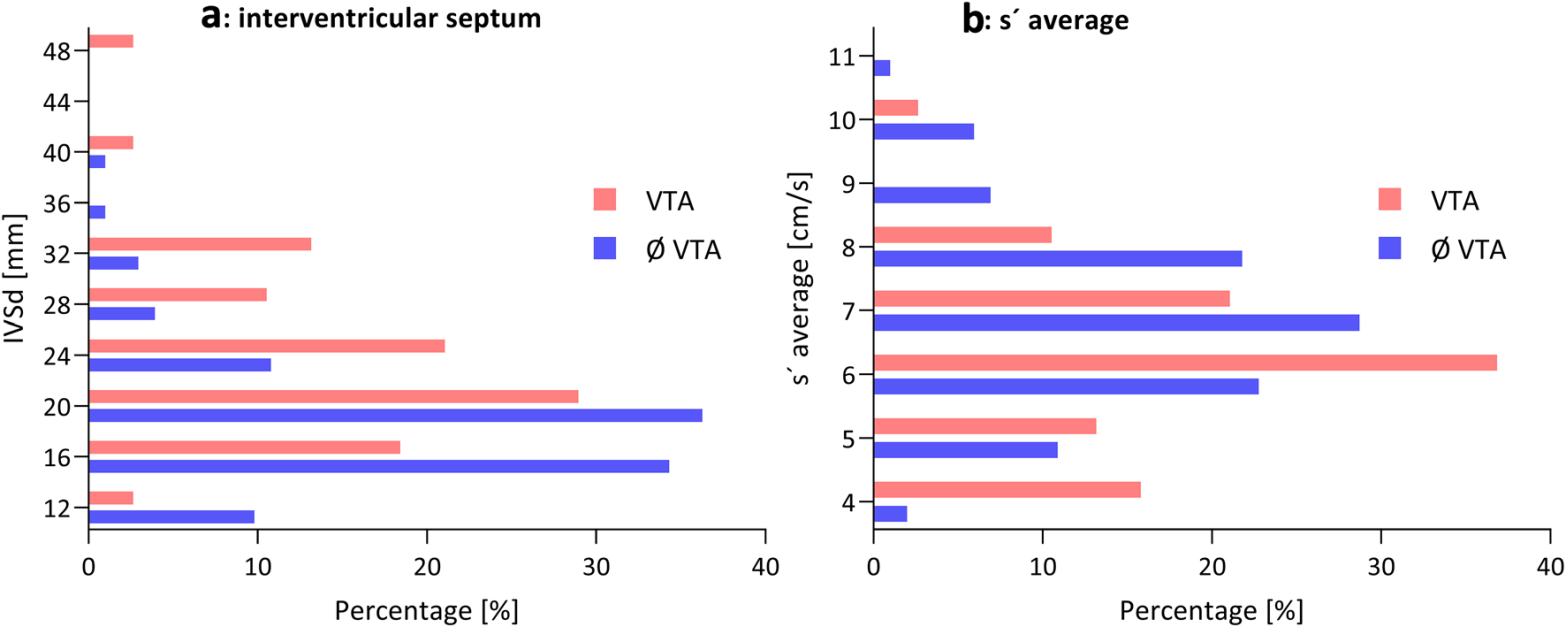
Histogram for IVSd (**a**) and s′ average (**b**) in our study cohort in VTA vs. no VTA groups. IVSd, interventricular septum in diastole; s′ average, averaged peak systolic longitudinal mitral annular velocity measured with Tissue Doppler imaging; VTA, ventricular tachyarrhythmia.

**Figure 3:**
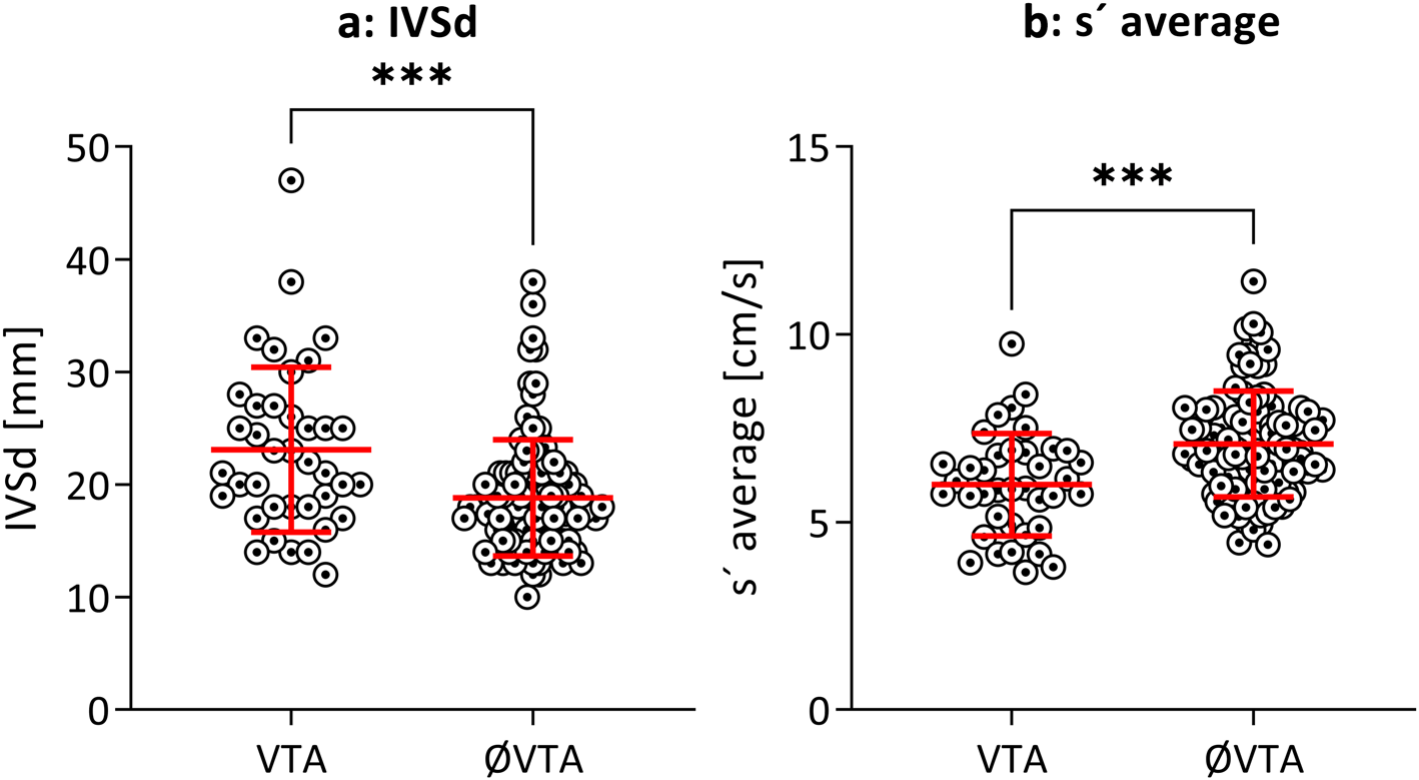
Thickness of interventricular septum (**a**) and s′ average (**b**) separated by the occurrence of VTA. IVSd, interventricular septum in diastole; s′ average, averaged peak systolic longitudinal mitral annular velocity measured with tissue doppler imaging; VTA, ventricular tachycardia.

2D echocardiography revealed a significantly thicker interventricular septum (22 [18–27] vs. 18 [15–21] mm; p < 0.001) in HCM patients with proven VTA (Figs. 2a, 3a). The risk of VTA increased with increasing IVSd. In contrast, the thickness of the posterior wall of the left ventricle (LVPWd) or the left ventricular end-diastolic diameter (LVEDD) did not differ significantly between the two groups (13 [10–13] vs. 12 [10–14] mm; p = 0.759 and 43 [40–49] vs. 42 [39–47] mm; p = 0.934, respectively). LAVI, which represents the size of the left atrium in relation to body surface area, was significantly increased in patients with VTA compared to those without (43 [37–72] vs. 39 [32–54] ml/m^2^; p = 0.015). On the other hand, LA PLAX did not differ between the two groups (p = 0.381), nor did mitral E wave, mitral A wave, E/A and deceleration time of the mitral E wave.

In tissue doppler imaging late diastolic velocity, a′ average (7.1 [5.8–9.1] vs. 8.9 [7.4–10.3] cm/s; p < 0.001, Table 1) and peak systolic velocity, s′ average (5.9 [4.9–6.9] vs. 6.9 [6.1–8.0] cm/s; p < 0.001, Table 1), were significantly reduced in patients with VTA when compared with patients without clinical endpoints (Figs. 2b, 3b). The risk of VTA increased with decreasing tissue doppler velocities. We could not make this observation for e′ average: the early diastolic velocity (5.9 [4.7–7.9] vs. 6.2 [4.8–7.7] cm/s; p = 0.758).

### Analysis for risk stratification

**Figure 4:**
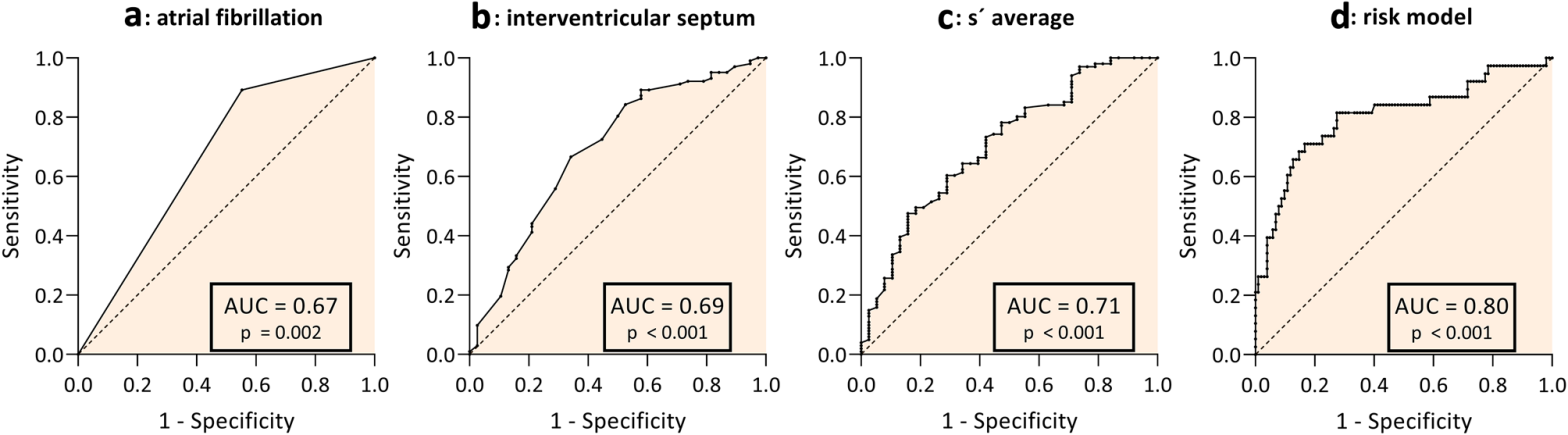
Receiver operating characteristic (ROC) curve to assess the ability of (**a**) atrial fibrillation, (**b**) interventricular septum, (**c**) s′ average and (**d**) the entire risk model (AF, s′ average, IVSd) to discriminate HCM patients with and without VTA.

Based on the evaluated parameters, s′ average was the parameter most strongly associated with the occurrence of VTA. Its discriminative ability was assessed by ROC analysis revealing an AUC of 0.71 (95% CI 0.61–0.81) (Fig. 4c). The ability of IVSd to discriminate for VTA occurrence was similar with an AUC of 0.69 (95% CI 0.59–0.80) (Fig. 4b). In addition, this parameter strongly correlated with the left ventricular mass index (r = 0.74; p < 0.001). Concerning AF, calculation of the ROC curve revealed an AUC of 0.67 (95% CI 0.56–0.78) (Fig. 4a). For s′ average, the logistic regression yields a negative coefficient implying that a higher value of s′ average decreases the probability of VTA occurrence. The corresponding ROC curve was inverted to facilitate comparison with the other parameters (Fig. 4c).

Table 2 shows the results of univariate and multivariate logistic regression analysis. History of atrial fibrillation (OR = 6.7; 2.7–16.4; p < 0.001), IVSd (OR = 1.12; 1.05–1.20; p < 0.001) and s′ average (OR = 0.54; 0.39–0.75; p < 0.001) were independently associated with VTA occurrence in univariate logistic regression. All three parameters remained significant in multivariate regression (OR = 5.5; 2.1–14.4; p < 0.001; OR = 1.09; 1.02–1.17, p = 0.014; OR = 0.58; 0.4–0.85; p = 0.005, respectively), even after adjustment for age and gender (OR = 6.1; 2.2–16.9, p < 0.001; OR = 1.09; 1.01–1.17 p = 0.023; OR = 0.59; 0.40–0.86 p = 0.006). The beta coefficients in the logistic equation correspond to the natural logarithm of the Odds ratio (ln(OR) = β; OR = e^β^).

**Table 2:** Univariate and multivariate logistic regression.

	Univariate logistic regression		Multivariate logistic regression		β-coefficient
	Odds ratio [95% CI]	p-value	Odds ratio [95% CI]	p-value	
Atrial fibrillation	6.7 (2.7–16.4)	< 0.001	5.5 (2.1–14.4)	< 0.001	1.702
IVSd per 1 mm increase	1.12 (1.05–1.20)	< 0.001	1.09 (1.02–1.17)	0.014	0.088
s′ average per 1cm/s increase	0.54 (0.39–0.75)	< 0.001	0.59 (0.40–0.85)	0.005	− 0.535
LAVI (ml/m^2^)	1.03 (1.01–1.04)	0.009			
a′ average per 1cm/s increase	0.76 (0.62–0.91)	0.004			

IVSd, interventricular septum in diastole; LAVI, Left atrial volume index; CI, confidence interval.

LAVI and a′ average missed statistical significance in multivariate logistic regression and are therefore not illustrated.

### Score development

A risk score for the occurrence of VTA was created using logistic regression based on data collected in routine clinical practice. Atrial fibrillation, the IVSd and s´ average were included in the final model since these parameters remained significant in multivariate logistic regression, even after adjustment for age and gender. The AUC was calculated to evaluate the discriminatory ability of the risk stratification score. The model demonstrated discrimination ability evidenced by an AUC of 0.80 (Fig. 4d). At a cutoff value of > 0.39; the model exhibited a sensitivity of 63% and a specificity of 88% in distinguishing HCM patients regarding the occurrence of VTA. Our risk stratification analysis revealed a positive predictive value of 67% and a negative predictive value of 87%.

The history of atrial fibrillation must be indicated in the score equation by assigning a value of 1 (apparent) or 0 (in-apparent), respectively. Additionally, the values of s′ average (in cm/s) and IVSd (in mm) must be inserted. The result of the logistic equation below is the probability (p) of the occurrence of VTA. If the score is > 0.39, this risk score permits the identification of patients with HCM at an increased risk for VTA and following intensified rhythm monitoring.

Probability (p) of the occurrence of VTA:

1 $$p=\frac{1}{1+{{\text{e}}}^{-z}}$$

$$z=0.231+1.702\cdot {\text{AF}}-0.535\cdot {s}^{{{\prime}}}\mathrm{ average }\left[\frac{cm}{s}\right]+0.088\cdot \mathrm{IVSd }[{\text{mm}}]$$

## Discussion

In this study, we developed a risk stratification model based on routine clinical parameters to identify HCM patients at increased risk for VTA. The proposed model is straightforward to implement and requires only a few components, such as ECG, two-dimensional echocardiography, and TDI, routinely acquired during patient visits. This model or score should facilitate the identification of patients who will require intensified rhythm monitoring in the future. During the course of the disease, numerous remodeling processes such as fibrosis, myocyte disarray and hypertrophy occur in the myocardium, affecting both the atrium and the ventricle^2,15^. Several studies have demonstrated that these remodeling processes increase the risk of cardiac arrhythmia in HCM patients^16,17^. Consequently, parameters indicating these remodeling processes in both the atrium and the ventricle were included in our analysis.

There is a correlation between atrial fibrillation and structural remodeling processes in the atrium such as fibrosis.^18^. A systematic review including more than 7000 HCM patients revealed an overall AF prevalence of 22.45%^19^, similar to ours (20%). Kubo et al. have shown that patients suffering from HCM and AF simultaneously developed more adverse cardiovascular events, including sustained ventricular tachycardia, as compared to AF-free HCM patients, assuming AF to be a trigger of adverse events^20^. Different studies came to a similar conclusion revealing AF as an independent predictor of ventricular tachyarrhythmia in HCM patients who underwent ICD implantation for primary prevention and recurrence of ventricular tachyarrhythmia in ICD recipients^21,22^. Patients suffering from atrial fibrillation usually show an enlarged left atrium^18^. For this reason, we also inspected the left atrial size in our study using two measurement methods. When analyzing LA PLAX, the diameter of the left atrium in the parasternal long axis, there were no variances regarding VTA. However, we were able to determine significant differences when examining LAVI, a value normalized to body surface area. In a study by Debonnaire et al. LAVI could even predict appropriate ICD therapy in patients with HCM^23^. Significant differences in our study cohort were observed for atrial fibrillation and LA size. However, an enlarged left atrium predisposes to atrial fibrillation, so the two parameters would be interdependent values^24,25^. Since predictors in risk stratification models should be independent of each other, we decided to use atrial fibrillation because of its stronger p-value. With the addition of this parameter in the risk score, the atrial remodeling processes should be properly represented.

Another parameter associated with the remodeling processes in the HCM myocardium is the IVSd. Our echocardiographic examination showed a strong correlation with the left ventricular mass index (r = 0.74), indicating the close association between IVSd and the extent of ventricular hypertrophy. Consequently, IVSd is an excellent parameter for estimating the degree of hypertrophy. In addition, this parameter has been identified as a measure of the severity of HCM and an important prognostic factor^26,27^. Several other studies have revealed that the presence of VTA is associated with the magnitude of left ventricular hypertrophy^10,27^. It was even identified as a predictor of SCD in HCM patients^9^ and was therefore included in the ESC risk score estimating the 5-year risk of SCD^4^. With the addition of this parameter to the risk model, the extent of hypertrophy is particularly taken into account.

A parameter reflecting long-axis systolic function and remodeling processes in the ventricle is the averaged peak systolic longitudinal mitral annular velocity (s′) measured with TDI. It was recently demonstrated that s′ average and diastolic mitral annular velocities could predict poor outcomes in patients suffering from various heart diseases^28–30^. The decrease in s′ can be explained by progressive fibrosis and remodeling in the myocardium: longitudinal myofibrils predominate in the subendocardium resulting in the contraction being dominant in the longitudinal direction, while the subepicardial contraction generates mainly circumferential shortening and twist^31^. Due to extreme fluctuations in pressure and compression and large distances to the epicardial coronary flow, which are even longer in hypertrophic hearts, the subendocardium is uniquely vulnerable to injury^31^. Therefore, the subendocardium is usually the first myocardial layer to show structural changes such as fibrosis and ischemia^31^. These fibrotic remodeling processes, which predominantly affect longitudinal myofibrils, can result in a reduced systolic function obtained with s′ while the LVEF remains unaffected. In general, the LVEF is not a suitable method to assess the systolic function in HCM patients since it is usually normal or even increased, although the systolic function is impaired^32,33^. By adding s′ average to the risk score, attention is paid to the left ventricle, its remodeling and general systolic function.

Our results were in line with different TDI studies. *Barakat *et al*.* reported the association between a reduced s′ with the occurrence of VTA or ventricular fibrillation irrespective of age, gender and LVEF in cardiac device recipients^34^. *Bayrak* et al. identified a lower lateral s′ associated with a higher risk of cardiovascular death and hospitalization due to worsening of heart failure symptoms in patients with HCM^35^. Similar to other studies^30,36^, we also detected a significant reduction in late mitral annular diastolic velocity (a′), which reflects the ventricles passive motion (Table 1). However, this value cannot be measured if the patient is currently in atrial fibrillation during the echocardiographic examination. Our goal was to include parameters in the prediction model that can be measured not only in sinus rhythm, especially since many HCM patients suffer from atrial fibrillation.^19^. Moreover, a′ is a value that initially increases in the course of progressing LV dysfunction, while s′ decreases directly^35^. Therefore, we decided to utilize s′ average as a parameter for the risk model.

In some studies, a significantly prolonged QTc interval in patients with VTA could be observed^7,8^. Similar to the mentioned studies, we also observed a tendency towards a longer QTc interval albeit without statistical significance.

Our study selected parameters that reflect both atrial and ventricular remodeling processes for our risk stratification model: atrial fibrillation, thickness of interventricular septum and s′ average. These parameters appear capable of predicting the occurrence of VTA in such patients due to their association with remodeling processes of the atrium and ventricle. The developed risk stratification model resulted in an accuracy of 81.4% in correctly diagnosing VTA in our study cohort. The sensitivity of the model was 63%, while the specificity was 88%. The positive predictive value was 67%, while the negative predictive value was 87%. Thus, a negative result from our risk stratification model (below the cut-off value) can be used to rule out VTA. The negative predictive value is of particular importance in clinical applications. Patients scoring above the cut-off value may benefit from extended rhythm monitoring to detect VTA.

Our study has some limitations. One limitation is the inclusion of data obtained from Holter ECG and ICD interrogation, resulting in differences in patient observation periods. This discrepancy results from the consideration of the last interrogation period for ICD interrogations. In addition, it should be noted that VTA detection criteria vary for ICD because of different programming approaches. Acceptance of this selection bias in our study was guided by our primary focus on exploratory assessment of VTA occurrence to elicit these same events through our model to identify patients at increased risk for VTA in the future. In addition, the cohort included three patients who had received treatment with antiarrhythmic drugs at one time in the patient history. Specifically, two individuals were administered flecainide and belonging to the non-VTA group, whereas one patient received amiodarone and belonging to the VTA group. The main limitation is the rather small number of patients, especially those who reached the endpoint. However, the proportion of patients with VTA is similar to that from other studies^37,38^. Although larger studies are needed to confirm our results in a validation cohort, we were able to make corroborated statements with our case number. Furthermore, the occurrence of VTA was retrospectively evaluated using an exploratory data set derived from a single center.

## Summary/conclusions

In summary, we have developed a risk stratification model using parameters that are easy to collect to detect HCM patients with an increased risk of ventricular tachyarrhythmia who consecutively require close rhythm monitoring. Independent prospective cohort studies should further validate our risk stratification model.

## Data Availability

The data sets generated in this study are available from the corresponding author upon reasonable request.

## Abbreviations

ACEi: Angiotensin converting enzyme inhibitor
AF: Atrial fibrillation
ARB: Angiotensin receptor blocker
AUC: Area under the curve
BMI: Body mass index
CCB DHP: Dihydropyridine calcium channel blocker
CCB non-DHP: Non-dihydropyridine calcium channel blocker
CI: Confidence interval
CMR: Cardiovascular magnetic resonance
ECG: Electrocardiogram
ESC: European Society of Cardiology
HCM: Hypertrophic cardiomyopathy
ICD: Implantable cardioverter defibrillator
IQR: Interquartile range
IVSd: Interventricular septum in diastole
LA PLAX: Left atrium in parasternal long axis
LAVI: Left atrial volume index
LGE: Late gadolinium enhancement
LVEDD: Left ventricular end diastolic diameter
LVEF: Left ventricular ejection fraction
LVPWd: Left ventricular posterior wall in diastole
MRA: Mineralocorticoid receptor antagonist
nsVT: Non-sustained ventricular tachycardia
NYHA: New York Heart Association
OR: Odds ratio
ROC: Receiver operating characteristic
SCD: Sudden cardiac death
SD: Standard deviation
sVT: Sustained ventricular tachycardia
TASH: Transcoronary ablation of septal hypertrophy
TDI: Tissue Doppler imaging
VTA: Ventricular tachyarrhythmia
